# Draft Genome Sequence of a *Halorubrum* H3 Strain Isolated from the Burlinskoye Salt Lake (Altai Krai, Russia)

**DOI:** 10.1128/genomeA.00566-15

**Published:** 2015-06-04

**Authors:** Aleksey S. Rozanov, Alla V. Bryanskaya, Tatiana K. Malup, Anastasia V. Kotenko, Sergey E. Peltek

**Affiliations:** Institute of Cytology and Genetics, Siberian Branch of the Russian Academy of Sciences, Novosibirsk, Russia

## Abstract

A *Halorubrum* H3 strain was isolated from a water and silt sample from Burlinskoye Lake (Altai Krai, Russia, 53°8′19″N 78°24′27″E). According to 16S rRNA sequences, this strain is most closely related to *Halorubrum saccharovorum*. The completely sequenced and annotated genome is 3,282,373 bp and contains 3,237 genes.

## GENOME ANNOUNCEMENT

The studied *Halorubrum* H3 strain was isolated from a water and silt sample taken from Burlinskoye (a salt lake in Altai Krai, Russia, 53°8′19″N 78°24′27″E). The water temperature was 20°С at the time of sampling.

The genus *Halorubrum* includes extremely halophilic archaeal species that are widely distributed in high-saline environments and are a major component of the microbial community of hypersaline environments (3 to 5 M) (1). The genus currently contains 26 species. Its representatives are widely distributed in hypersaline environments on almost all continents (2–6).

*Halorubrum* H3 culture was cultivated in liquid medium containing 1% tryptone, 0.5% yeast extract, and 3.5 M NaCl. Eight milliliters of cell culture was pelleted by centrifugation and resuspended in 75 µl of H_2_O by intense pipetting. DNA was isolated using the DNA purification kit (Fermentas). The Ion PGM Template OT2 400 kit was used to create libraries for genome sequencing. Genome sequencing was performed on an Ion Torrent platform (Applied Biosystems) with Ion 316 chip using the Ion PGM sequencing 400 kit at the Siberian Branch of the Russian Academy of Sciences (SBRAS) Sequencing Center.

*De novo* assembly of short reads into contigs was performed using SPAdes 3.1.0. Contigs <1,000 bp were deleted. A total of 189 contigs yielded a genome sequence 3,282,373 bp long, with a G+C content of 67.57%. Open reading frame (ORF) prediction and automatic annotation were performed using the NCBI Prokaryotic Genome Annotation Pipeline (http://www.ncbi.nlm.nih.gov/genomes/static/Pipeline.html). The complete genome sequence contains 3,237 genes, 2,337 coding sequences (CDSs), 3 rRNAs (5S, 16S, and 23S), 46 tRNAs, and 1 noncoding RNA (ncRNA).

Phylogenetic analysis was performed using 16S rRNA sequences with the unweighted pair group method using average linkages (UPGMA) algorithm implemented in MEGA version 6. The 16S rRNA sequences of *Halorubrum* type strains were found using the StrainInfo (http://www.straininfo.net) and GenBank (http://www.ncbi.nlm.nih.gov/nucleotide) databases. According to phylogenetic analysis, the *Halorubrum* H3 strain is most closely related to *Halorubrum saccharovorum*.

### Nucleotide sequence accession numbers. 

The draft genome sequence for *Halorubrum* H3 has been deposited in GenBank under the accession number JNFH00000000. The 189 contigs have been deposited under the accession numbers JNFH02000001 to JNFH02000189.
